# Molecular characterization of three intestinal protozoans in hospitalized children with different disease backgrounds in Zhengzhou, central China

**DOI:** 10.1186/s13071-019-3800-5

**Published:** 2019-11-15

**Authors:** Fuchang Yu, Dongfang Li, Yankai Chang, Yayun Wu, Zhenxin Guo, Liting Jia, Jinling Xu, Junqiang Li, Meng Qi, Rongjun Wang, Longxian Zhang

**Affiliations:** 1 College of Animal Science and Veterinary Medicine, Henan Agricultural University, Zhengzhou, Henan People’s Republic of China; 2 National Joint Research Center for Animal Immunology, Zhengzhou, Henan People’s Republic of China; 3 Zhengzhou Children’s Hospital, Zhengzhou, Henan People’s Republic of China; 4 The Third Affiliated Hospital of Zhengzhou University, Zhengzhou, Henan People’s Republic of China; 5 Henan Province People’s Hospital, Zhengzhou, Henan People’s Republic of China; 6 Scientific Research Experiment Center & Laboratory Animal Center, Henan University of Chinese Medicine, Zhengzhou, 450046 People’s Republic of China; 7 College of Animal Science, Tarim University, Alar, Xinjiang People’s Republic of China

**Keywords:** *Cryptosporidium*, *Giardia duodenalis*, *Enterocytozoon bieneusi*, Epidemiology, Children, China

## Abstract

**Background:**

*Cryptosporidium* spp. and *Giardia duodenalis* are major intestinal pathogens that can cause diarrheal diseases in humans, especially children. *Enterocytozoon bieneusi* is another parasite which can cause gastrointestinal tract disorders, with diarrhea being the main clinical symptom. However, few genetic studies of these parasites in pediatric inpatients in China have been published.

**Methods:**

To assess the genetic characteristics and epidemiological status of these parasites, a total of 2284 fecal samples were collected from children in the pediatric departments of three hospitals in Zhengzhou, central China, and screened for these protozoans with PCR, based on the small subunit ribosomal RNA (*SSU* rRNA) genes of *Cryptosporidium* spp. and *G. duodenalis* and the internal transcribed spacer (ITS) of *E. bieneusi*.

**Results:**

Six (0.26%), 14 (0.61%), and 27 (1.18%) of the samples were positive for *Cryptosporidium* spp., *G. duodenalis* and *E. bieneusi*, respectively. Of the 12 successfully sequenced *G. duodenalis* isolates, four were identified as assemblage A and eight as assemblage B. In subtype and multilocus genotype (MLG) analyses, *C. parvum* IIdA19G1 (*n* = 4) and two novel *G. duodenalis* MLGs belonging to subassemblage AII (*n* = 3) and BIV (*n* = 5) were successfully identified. The *E. bieneusi* isolates included genotypes D (*n* = 17), J (*n* = 2), PigEBITS7 (*n* = 1), BEB6 (*n* = 1), and CM8 (*n* = 1). This is the first report of *C. parvum* subtype IIdA19G1 in HIV-negative children and *E. bieneusi* genotype CM8 in humans.

**Conclusions:**

The dominance of zoonotic *C. parvum* subtype IIdA19G1 indicates that this parasite is turning into zoonotic origin from human-to-human transmission. The phylogenetic analysis also revealed the zoonotic origins and anthroponotic transmission potential of *G. duodenalis* and *E. bieneusi*, suggesting more efforts must be made to minimize the threat these pathogens pose to public health.
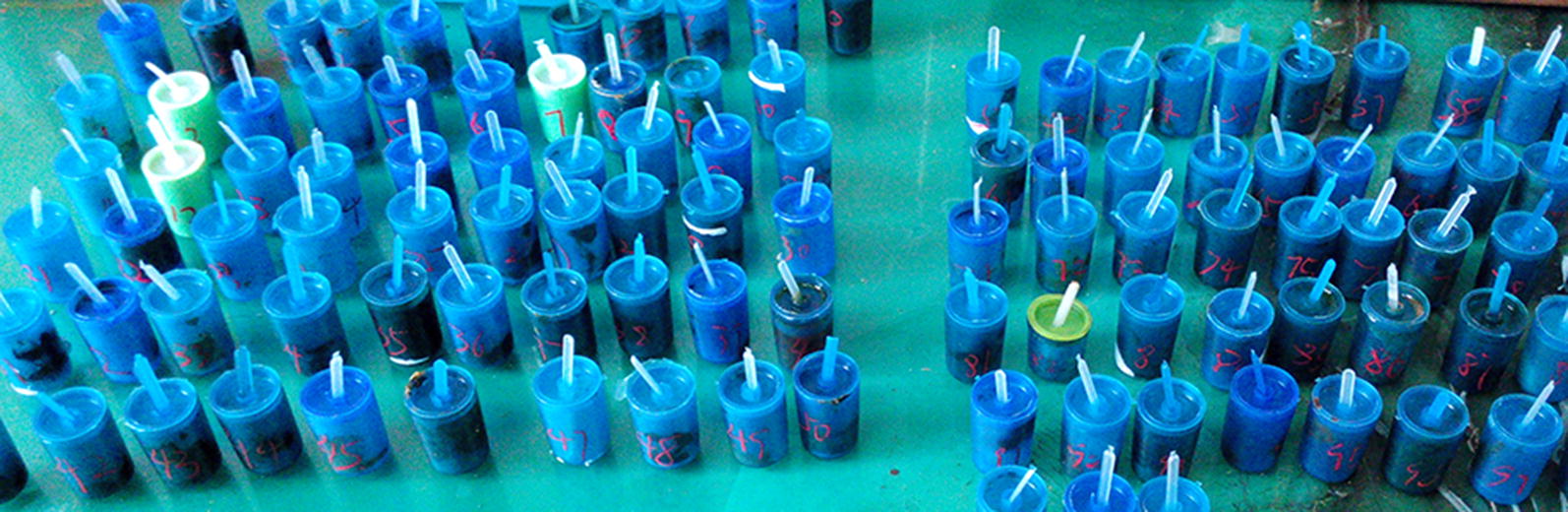

## Background

*Cryptosporidium* spp., *Giardia duodenalis* and *Enterocytozoon bieneusi* are the major etiological agents of cryptosporidiosis, giardiasis and microsporidiosis, respectively [1–3]. When infected with these parasites, although most of the immunocompetent individuals are always asymptomatic, there are also patients suffering self-limiting diarrhea or severe wasting disease, especially immunocompromised individuals, such as HIV-positive patients [4, 5]. Susceptible hosts acquire infections through both direct and indirect transmission routes, such as direct contact or ingestion of contaminated food or water [5].

Molecular diagnostic tools based on the small subunit ribosomal RNA (*SSU* rRNA) gene of *Cryptosporidium* spp. and *G. duodenalis* and the internal transcribed spacer (ITS) of *E. bieneusi* have long been used for the molecular analysis of these parasites [3]. At least 36 valid species of *Cryptosporidium* have been reported worldwide [6], among which *C. hominis* and *C. parvum* are the *Cryptosporidium* species most frequently detected in human isolates [3]. *Giardia duodenalis* is considered a species complex, consisting of eight genetically distinct genotypes, assemblages A-H, which have different host specificities. Assemblages A and B appear to be responsible for most human infections [7, 8]. Currently, over 1600 *E. bieneusi* ITS genotypes have been deposited in GenBank, derived from more than 650 studies [9]. Of these, genotypes D, EbpC, PigEBITS7 and type IV, are the genotypes most prevalent in humans [9].

According to the molecular epidemiological data available from many provinces in China (Jilin, Heilongjiang, Tianjin, Anhui, Hebei, Henan, Jiangsu, Shanghai, Hubei, Sichuan, Chongqing, Guangxi and Yunnan) [5, 10–12], the prevalence of *Cryptosporidium* ranges from 0.1% to 13.5% [13, 14]. Epidemiological investigations conducted in recent years have suggested that the average infection rate of *G. duodenalis* is 0.85% (197/23,098), and the highest infection rate (9.46%, 7/74) was reported in one study in Shanghai [10, 15]. The prevalence of *E. bieneusi* in humans varies from 0.2% to 22.5%, with the highest infection rate observed in children in Jilin Province [11, 16].

Most published studies of these parasites have involved HIV-positive patients and children with diarrhea, but the infectious status of these parasites in children with different disease backgrounds remains unclear. Henan is a province located in central China, with a population of about 100 million. As the capital of Henan Province, Zhengzhou, has a population of more than 10 million and the public health threat in the event of an outbreak of these parasitic diseases is significant. Because the patients hospitalized in pediatric wards are by definition children, outbreaks of enteroparasites are more likely to occur when these children infected with highly virulent isolates. Therefore, we thoroughly assessed the prevalence and risk levels of *Cryptosporidium* spp., *G. duodenalis* and *E. bieneusi* in a large population of children hospitalized in Zhengzhou, Henan Province, central China.

## Methods

### Sample collection

Hospitals are ideal sites for surveying individuals with different disease backgrounds, and fecal samples can be readily collected. In this study, the pediatric departments of three hospitals in Zhengzhou, Henan Province, were selected to investigate the infection rates and genetic characteristics of *Cryptosporidium*, *G. duodenalis* and *E. bieneusi* in the children admitted to these hospitals.

All the parents or guardians were given specific oral instructions for the proper sample collection and the avoidance of possible contamination, and a plastic box labeled with a unique number in which to collect a fresh stool sample in the morning. A total of 2284 samples were collected from children aged 1–14 between March 2016 and January 2017, and all the samples were stored at 4 °C in 2.5% (w/v) potassium dichromate solution until DNA extraction.

### DNA extraction and PCR amplification

Dichromate was washed off the samples by distilled water and centrifugation. Genomic DNA was extracted from ~200 mg of each stool sample using the E.Z.N.A^®^ stool DNA kit (Omega Bio-tek, Norcross, GA), according to the manufacturer’s instructions. Extracted DNA preparations were analyzed by nested PCR amplification.

All samples were examined for *Cryptosporidium* spp. using a nested PCR that targeting a ~834-bp fragment of the *SSU* rRNA gene [17, 18] The *Cryptosporidium*-positive samples were further analyzed with nested PCR targeting a 400–500-bp fragment of the 60 kDa glycoprotein gene (*gp60*) [19]. All the samples were tested for *G. duodenalis* infection with nested PCR based on a ~292-bp fragment of the *SSU* rRNA, using a previously described assay [20]. Segments of the β-giardin gene (*bg*, ~510 bp), triose phosphate isomerase gene (*tpi*, ~530 bp) and glutamate dehydrogenase gene (*gdh*, ~530 bp) were then amplified with the method described by Sulaiman et al. [21], Cacciò et al. [22] and Lalle et al. [23] to determine the multilocus genotypes (MLGs) of the *G. duodenalis* isolates detected in this study. For the assemblage-specific amplification of the Giardia *tpi* gene, primers previously described by Geurden et al. [24] were used. The presence of *E. bieneusi* in the 2284 fecal samples was detected with nested PCR that amplified a ~389-bp fragment of the ITS [25].

Reagent-grade water was used as the negative control for all PCRs and DNA known to be positive at the specific locus was used as the positive control. The amplified fragments were separated electrophoretically on 1% agarose gel stained with DNAGreen (Tiandz, Beijing, China) and visualized under UV light.

### Sequence and data analyses

The positive secondary PCR products were sequenced bidirectionally on an ABI PRISM™ 3730 XL DNA Analyzer by SinoGenoMax Biotechnology Co., Ltd (Beijing, China) using the BigDye Terminator v3.1 Cycle Sequencing Kit (Applied Biosystems, Foster City, CA, USA). The generated sequences were assembled and edited with DNASTAR Lasergene EditSeq version 7.1.0 (http://www.dnastar.com/) and aligned with reference sequences downloaded from GenBank with Clustal X version 2.1 (http://www.clustal.org/).

To infer the phylogenetic relationships of the detected samples, neighbor-joining (NJ) trees were constructed with the program MEGA X (http://www.megasoftware.net), based on evolutionary distances calculated with the Kimura 2-parameter model. The reliability of these trees was assessed with a bootstrap analysis of 1000 replicates.

All statistical analyses were performed with the software IBM SPSS Statistics (www.ibm.com/products/spss-statistics). The prevalence of parasitic infections, with the 95% confidence interval (CI), was calculated. In the univariate analyses, Fisher’s exact test was used to compare the prevalence of the three parasitic diseases in groups constructed according to age, sex, and disease background, and the odds ratios (ORs; with 95% CIs) were computed to examine the associations between the participants’ characteristics and the parasitic infections A two-sided *P*-value of 0.05 or less was deemed significant.

## Results

### Occurrence of *Cryptosporidium* spp., *G. duodenalis* and *E. bieneusi*

Of the 2284 fecal samples examined in this study from the three children’s hospitals in Zhengzhou, six (0.26%, 95% CI: 0.03–0.49%), 14 (0.61%, 95% CI: 0.27–0.95%), and 27 (1.18%, 95% CI: 0.72–1.65%) were positive for *Cryptosporidium* spp., *G. duodenalis* and *E. bieneusi*, respectively (*P* = 0.0007). No coinfections of these pathogens were detected in any of the samples. By age, the highest total infection rate (5.22%, 14/268, 95% CI: 2.37–8.07%) for all three parasites combined was detected in children aged 6–14 years, followed by children aged 1–6 years, with an infection rate of 2.48% (23/927, 95% CI: 1.43–3.54%); the lowest rate was 0.92% (10/1089, 95% CI: 0.31–1.53%) in children aged < 1 year (*P* < 0.0001) (Table 1). The data showed that 2.12% (95% CI: 1.33–2.90%) of males and 1.96% (95% CI: 0.98–2.94%) (*P* = 0.88) of females were positive for an intestinal parasite. According to the children’s disease backgrounds, the highest infection rate (8.33%, 13/156, 95% CI: 3.68–12.99%) was observed in the group with gastrointestinal symptoms, followed by the group with autoimmune rheumatic disease (4.98%, 11/221, 95% CI: 1.88–8.07%) or hematological neoplasms (4.14%, 6/145, 95% CI: 0.55–7.72%), whereas only 0.96% (17/1762, 95% CI: 0.48–1.45%) of the remaining 1762 samples from children with other medical conditions were positive (*P* < 0.0001) (Table 1).

**Table 1:** Occurrence of three intestinal protozoans in children by age, sex and disease background

Variable	No. of participants	Prevalence (95% CI) (%)	*P*-value	OR (95% CI)
Age (years)				
< 1	1089	0.92 (0.31–1.53)	< 0.0001	1.00
1–6	927	2.48 (1.43–3.54)	0.007	2.75 (1.30–5.80)
6–14	268	5.22 (2.37–8.07)	< 0.0001	5.97 (2.61–13.54)
Sex				
Male	1416	2.12 (1.33–2.90)		1.00
Female	868	1.96 (0.98–2.94)	0.88	0.92 (0.51–1.68)
Disease background				
Other disease	1762	0.96 (0.48–1.45)	< 0.0001	1.00
Gastrointestinal disease	156	8.33 (3.68–12.99)	< 0.0001	9.33 (4.44–19.60)
Autoimmune rheumatic disease	221	4.98 (1.88–8.07)	< 0.0001	5.38 (2.49–11.63)
Hematology neoplastic disease	145	4.14 (0.55–7.72)	0.006	4.43 (1.72–11.42)

### Correlation analysis

The correlations between the clinical data and the infection rates were evaluated by computing the ORs and their 95% CIs, which are shown in Table 1. There was a significant positive correlation between the infection rate and age in the hospitalized children, as an OR of 2.75 (95% CI: 1.30–5.80; *P* = 0.007) was associated with the 1–6 year-old group and 5.97 (95% CI: 2.61–13.54, *P* < 0.0001) was associated with the 6–14 year-old group. No significant association between infection and sex (OR = 0.92, 95% CI: 0.51–1.68) was observed. The results of the univariate analysis also showed that children with gastrointestinal symptoms (OR = 9.33, 95% CI: 4.44–19.60), autoimmune rheumatic disease (OR = 5.38, 95% CI: 2.49–11.63), or hematological neoplasms (OR = 4.43, 95% CI: 1.72–11.42) were at greatest risk of parasitic infection.

### *Cryptosporidium* species and *gp60* subtypes

The sequences of the *SSU* rRNA gene were obtained for five of the six positive samples, which showed that four of the patients were infected with *C. parvum* and one with *C. hominis*. All four *C. parvum* samples were subtyped as IIdA19G1R1, with no heterogeneity, whereas the single *C. hominis* sample was not typed successfully at the *gp60* locus (Table 2).

**Table 2:** Occurrence and subtype, assemblage, or genotype distributions of *Cryptosporidium* spp., *G. duodenalis* and *E. bieneusi* detected in this study

Parasites	Prevalence (%)	Subtype/assemblage/genotype^a^
*Cryptosporidium* spp.	0.26 (*n* = 6)	IIdA19G1 (*n* = 4)
*G. duodenalis*	0.61 (*n* = 14)	Assemblage A (*n* = 4); assemblage B (*n* = 8)
*E. bieneusi*	1.18 (*n* = 27)	D (*n* = 17); J (*n* = 2); PigEBITS7 (*n* = 2); BEB6 (*n* = 1); CM8 (*n* = 1)

^a^Not all of the positive samples were sequenced successfully

### *Giardia duodenalis* assemblages and multilocus genotypes

Of the 14 PCR-positive samples of *G. duodenalis* at the *SSU* rRNA locus, 12 were sequenced successfully. However, four were identified as assemblage A and eight as assemblage B. When the MLG technique was applied to these 14 samples, 11, 10 and 12 samples were positive at the *bg*, *tpi* and *gdh* loci, respectively (Tables 2, 3).

A sequence analysis of the 11 sequences positive at the *bg* locus showed that five isolates were assemblage A and six were assemblage B. The sequences from all the assemblage A isolates were identical to GenBank reference sequence MG736240. Within the assemblage B isolates, three sequences were identical to MG736242, whereas two sequences had a single-nucleotide polymorphism (SNP) (G389A or G239A) relative to the MG736242 sequence, and the remaining sequence had two SNPs (A95G and C188T).

According to the sequence analysis of the *gdh* locus, four isolates were identified as assemblage A and seven as assemblage B. Isolate ED551 showed inconsistent bidirectional sequencing results, because the forward sequence identified it as assemblage B, whereas the reverse sequence identified it as assemblage A. All four assemblage A sequences and the reverse sequence of isolate ED551 were consistent with KT948091. Apart from the forward sequence of isolate ED551, the sequences obtained for the assemblage B isolates were identical to EU637585, except one sequence with one SNP (T318C) relative to MH475908.

At the *tpi* locus, assemblages A and B were identified in four and six PCR-positive samples, respectively. One and three assemblage A sequences were identical to reference sequences MH673809 and MH673818, respectively. Among the assemblage B sequences, two sequences were consistent with KT948104 or KX668308, and the remaining four sequences showed one SNP (G250A) relative to KF679742. Because of its inconsistent bidirectional sequencing results at the *gdh* locus, isolate ED551 was amplified and sequenced again using assemblage A and assemblage B-specific primers of the *tpi* gene, respectively. Interestingly, a mixed infection of assemblage A and B was detected in ED551.

**Fig. 1:**
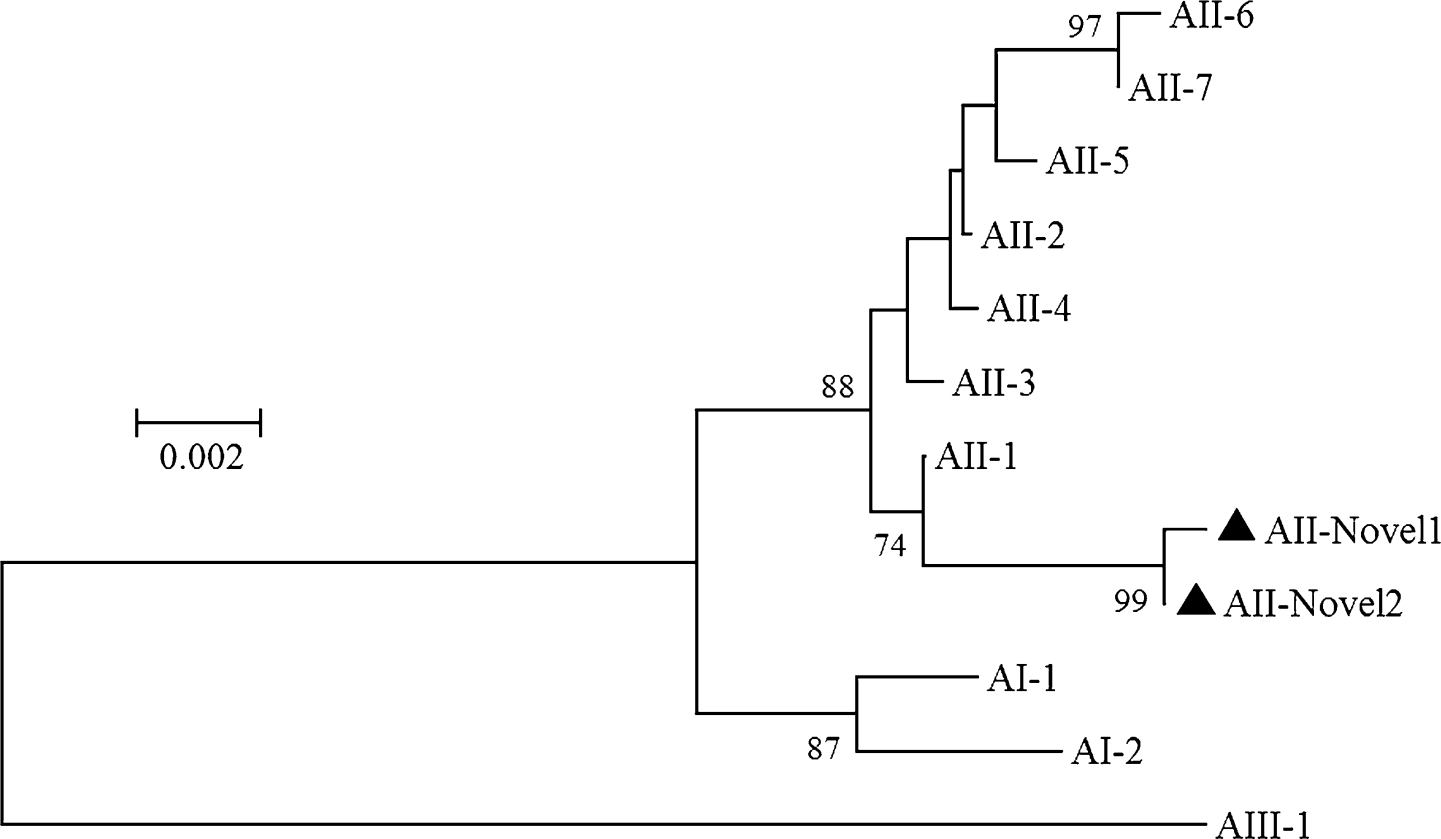
Phylogenetic relationships among *G. duodenalis* assemblage A isolates inferred with a neighbor-joining analysis based on the concatenated *bg*, *tpi* and *gdh* nucleotide sequences. Multilocus genotypes from previous studies were used as the reference sequences and are represented by the corresponding isolate name [22]. Novel MLGs are indicated with triangles. Bootstrap values > 70% from 1000 replicates are shown at the nodes

**Fig. 2:**
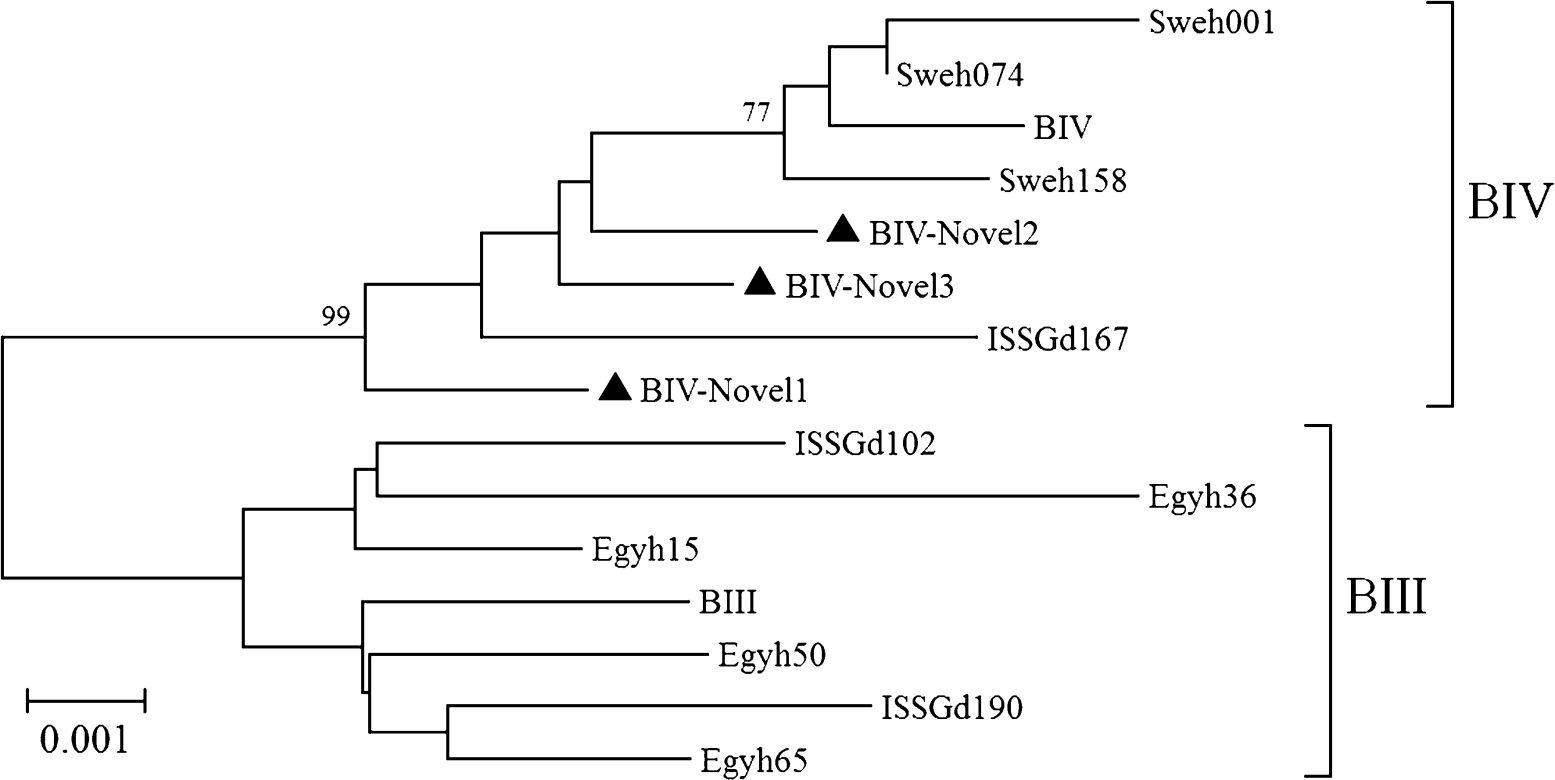
Phylogenetic relationships among *G. duodenalis* assemblage B isolates inferred with a neighbor-joining analysis based on the concatenated *bg*, *tpi* and *gdh* nucleotide sequences. Multilocus genotypes from previous studies were used as the reference sequences and are represented by the corresponding isolate names [8, 22, 47]. Novel MLGs are indicated with triangles. Bootstrap values > 70% from 1000 replicates are shown at the nodes

Because of its inconsistent bidirectional sequencing results, ED551 was excluded from the MLG analysis, as were those samples for which data for all three loci were not available. In total, five MLGs were generated (two belonging to assemblage A and three belonging to assemblage B) and were designated AII-Novel1, AII-Novel2, and BIV-Novel1 to BIV-Novel3. All the MLGs were found in a single isolate, except BIV-Novel3, which occurred in three isolates (Table 3). In a phylogenetic analysis, the two assemblage A MLGs clustered closely with MLG AII-1 (Fig. 1) and the three assemblage B MLGs clustered on the MLG BIV branch (Fig. 2).

**Table 3:** Multilocus genotyping of *Giardia duodenalis* isolates with a sequencing analysis of *SSU* rRNA, *bg*, *tpi* and *gdh* genes

Isolate	*G. duodenalis* assemblage				MLGs
	*SSU* rRNA	*bg*	*tpi*	*gdh*	
FY86	B	B	B	B	BIV-Novel1
FY120	SN	A	PN	B	
ET91	B	B	B	B	BIV-Novel2
ET129	B	B	B	B	BIV-Novel3
ET131	B	B	B	B	BIV-Novel3
ET265	A	A	A	A	AII-Novel1
ET346	A	A	A	A	AII-Novel2
ED127	B	B	B	B	BIV-Novel3
ED158	B	PN	B	PN	
ED221	B	B	PN	PN	
ED303	A	A	SN	A	
ED421	A	A	A	A	AII-Novel2
ED462	SN	PN	PN	B	
ED551	B	SN	A	A+B^a^	

*Abbreviations:* PN, PCR-negative; SN, sequence-negative

^a^Results of forward sequencing showed assemblage B, whereas reverse sequencing showed assemblage A

### *Enterocytozoon bieneusi* genotypes

**Fig. 3:**
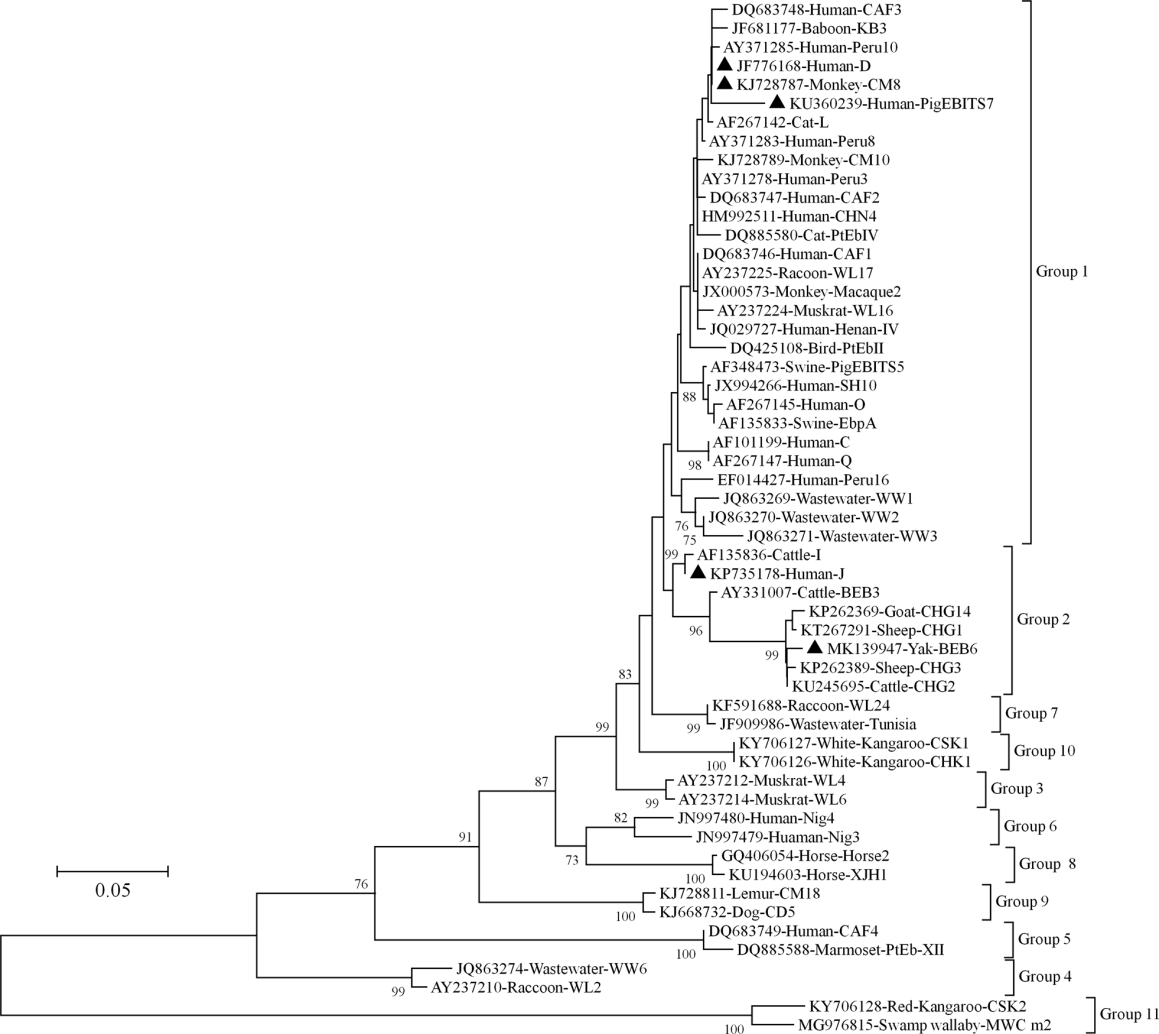
Neighbor-joining tree of *E. bieneusi* ITS genotypes. The phylogenetic relationships of the *E. bieneusi* genotypes determined in this study and other genotypes previously deposited in GenBank were inferred with a neighbor-joining analysis of ITS sequences based on genetic distances calculated with the Kimura 2-parameter model. Each sequence from GenBank is identified by its accession number, host origin, and genotype designation. Genotypes identified in this study are indicated with triangles. Bootstrap values > 70% from 1000 replicates are shown at the nodes

Of the 27 samples positive for *E. bieneusi* according to PCR amplification of the ITS fragment, 23 were sequenced successfully. Sequence analysis identified five genotypes: D, J, PigEBITS7, BEB6 and CM8. D was the dominant genotype and was detected in 17 isolates, whereas the other genotypes were restricted to one or two isolates (Table 2). In a phylogenetic analysis, three of the five genotypes identified in this study (D, CM8 and PigEBITS7) clustered in group 1 and genotypes J and BEB6 clustered in group 2 (Fig. 3).

## Discussion

Our data indicate that children aged 6–14 years were more likely to be infected with enteroparasites than those aged < 1 year or 1–6 years, suggesting that children aged 6–14 years are at highest risk of infection by these parasites, which is inconsistent with previous studies [26, 27]. These discrepancies might reflect the socioeconomic levels of the study sites, the immune status of the targeted groups, and/or the sensitivity of the techniques used [28]. In this study, newborns under one month of age were also included as long as they met the criteria. In the traditional Chinese concept of infant care, these newborns have little contact with anything other than their mothers during the first month of their lives. In one of the three hospitals involved in this study, the majority of pediatric inpatients were neonates, among whom no parasitic infections were found, and this may be contributed to the low infection rates of these parasites. Interestingly, higher infection rates were found in children with gastrointestinal symptoms, autoimmune rheumatic disease, or hematological neoplasms than in children with other diseases, which might be explained by the nature of these parasites, which cause gastroenteric symptoms, especially in immunocompromised patients [1–3]. The innate immune response plays an important role in the pathogenesis of autoimmune rheumatic disease, and many inhibitors are used today to treat this disease, including interleukin 6 (IL-6) inhibitors, IL-1 inhibitors, Janus kinase inhibitors and anti-B cell drugs [29]. Therefore, the immune function of the patient is inhibited to some extent, which may explain the high infection levels in the children with autoimmune rheumatic disease.

The prevalence of *Cryptosporidium* spp. (0.26%) was consistent with previous molecular epidemiological studies of *Cryptosporidium* in children in other cities and provinces of China (1.2% in Taiwan, 0.1% in Kunming, 2.0% in Wuhan, and 1.6% in Shanghai) [13, 30–32]. In a previous study conducted in Henan, the infection rate of *Cryptosporidium* was 1.5%, but it was 0.15% in 683 HIV-positive patients in the National Free Antiretroviral Therapy Programme (NFATP) in China [33]. *Cryptosporidium* was detected in 0.11% of the samples in a cross-sectional study of 1637 children aged 3–9 years in Jiangsu [28]. In Sichuan Province, the infection rate of *Cryptosporidium* was reported to be 2.4% in a school-based cross-sectional study involving 321 elementary school children [34].

In our sequence analysis of the positive PCR products, *C. parvum* predominated, and was detected in four of the five successfully sequenced *Cryptosporidium*-positive samples. Inconsistent results have been published in previous studies. In a genetic characteristic analysis conducted in Henan, *C. hominis* was identified in nine of 10 *Cryptosporidium* spp. isolates from human samples [35]. Similarly, *C. hominis* accounted for 90.2% (92/102) of *Cryptosporidium* spp. infections in patients in Shanghai [31]. When subtyped at the *gp60* locus, all the four *C. parvum* isolates detected in the present study were identified as IIdA19G1, a subtype which has recently been reported in several studies of human samples worldwide. For example, two *C. parvum* isolates derived from HIV-positive patients in Henan, China, were identified as IIdA19G1 [33]. This subtype has also been reported in human samples in Madrid, Spain [36] and in Portugal [37]. IIdA19G1 is also one of the most commonly detected subtypes in calves in Henan, Hebei, Beijing, and Heilongjiang Provinces [38–42] in horses in Sichuan, and in donkeys in Henan and Shandong Provinces [43]. *Cryptosporidium parvum* has been reported more frequently than *C. hominis* in most recent studies in China, suggesting a zoonotic origin rather than its anthroponotic transmission. This increasingly dominant transmission route of this parasite might be attributable to the rapid development of intensive breeding in recent decades and has important implications for public-health strategies to control these parasites.

In the present study, the infection rate of *G. duodenalis* was 0.61%. A large number of epidemiological investigations have been conducted in China since the start of this century, and have suggested that the average infection rate of *G. duodenalis* is 0.85% (197/23,098) [10]. Our data are consistent with a more recent study in Wuhan, where a low infection rate (1.4%) of *G. duodenalis* was detected [32]. In another study, *G. duodenalis* was detected in 17 of 252 fecal samples collected from patients with diarrhea in Shanghai, with an infection rate of 6.75%, which is higher than that in the present study [44]. A similar observation was also made in India, where a high rate of *G. duodenalis* infection (10.2%, 413/4039) was seen throughout the study period across all climatic conditions [45]. The infection rate of *G. duodenalis* is considered to be influenced by many factors, including ecological, environmental, and demographic conditions, and especially the immune status of the host [46].

In the MLG analysis of the *G. duodenalis* isolates, five MLGs were detected when the sequences at the *bg*, *tpi* and *gdh* loci were combined. In a phylogenetic analysis, two MGLs belonged to subassemblage AII and the remaining five MLGs clustered with subassemblage BIV. Subassemblages AII and BIV are the MLGs found most frequently in human samples in China [1, 15, 32, 35], suggesting an important role for anthroponotic transmission in the epidemiology of giardiasis. BIV was the most prevalent MLG in the present study, identified in 62.5% (5/8) of the *G. duodenalis* isolates, whereas BIII was found in none. However, different results have been observed in one of our previous study [47] and in a study conducted by Naguib et al. [48], in which an equal proportion of subassemblage BIII and BIV MLGs were reported. These discrepant results confirm the geographic segregation of the distributions of subassemblages BIII and BIV.

Of the fecal samples tested with PCR, 27 (1.18%) were positive for *E. bieneusi* infection. In China, the prevalence of *E. bieneusi* in humans varies from 0.2% to 22.5% [16, 32, 33, 49], and a similar infection rate (1.3%, 5/381) was detected in a previous study, in which 381 stool samples from cancer patients in China were screened [50]. As well as the immune status of the host, *E. bieneusi* infection rates are associated with many factors, including but not limited to the economic status and living conditions of the subjects [50].

In an analysis of the ITS sequences of *E. bieneusi*, D was the most common genotype, detected in 17 samples. According to the few reports of this parasite in humans in China, genotype D has been detected in children with diarrhea, HIV-positive patients, and HIV-negative individuals in Shanghai, Henan, Wuhan and Guangxi [15, 32, 33, 51]. Genotype D has also been detected in at least 15 animal species, including companion animals, livestock, wildlife, rodents, and birds [9, 50]. In one of our recent studies, this genotype was found in synanthropic rodents living on a dairy farm [52]. Genotype D has also been observed in river water and wastewater [9], and these data draw a concise profile of the zoonotic potential of *E. bieneusi* genotype D, which is transmitted *via* the indirect fecal-oral route. Genotypes PigEBITS7, J, and BEB6 may also be transmitted between humans and animals, because many researchers have reported human infections of these genotypes in China, which were first detected in animal samples [15, 16, 33, 51]. Interestingly, to the best of our knowledge, this is the first report of a human infection of genotype CM8, which was first found in non-human primates by Karim et al. [53]. It has since been shown to infect dairy cattle [54] and horses [55]. These data extend the host range of this genotype and demonstrate its zoonotic potential.

## Conclusions

We have shown low burdens of *Cryptosporidium* spp., *G. duodenalis* and *E. bieneusi* in children hospitalized in the pediatric departments of three hospitals in Zhengzhou, central China. A univariate analysis showed that children aged 6–14 years are most likely to be infected by these parasites, as are children with gastrointestinal symptoms, autoimmune rheumatic disease, or hematological neoplasms. The dominance of *C. parvum* subtype IIdA19G1 suggests the zoonotic origin of this parasite in children involved in this study. The occurrence of *G. duodenalis* subassemblages AII and BIV suggests the anthroponotic transmission of *G. duodenalis* and confirms the geographic segregation in the distributions of the different *G. duodenalis* subassemblages. The five *E. bieneusi* genotypes identified here have all been isolated from humans and a vast range of animal species, indicating that the zoonotic transmission of *E. bieneusi* is possible. Therefore, more holistic and integrated approaches must be used to minimize the threat posed by these parasitic pathogens to public health.

## Data Availability

Data supporting the conclusions of this article are included within the article. The newly generated sequences were deposited in the GenBank database under the accession numbers MK962813–MK962826, MK990042, MK990043 and MK990733–MK990737.

## Abbreviations

SSU rRNA: small subunit ribosomal RNA
ITS: internal transcribed spacer
HIV: human immunodeficiency virus
*gp60*: the 60 kDa glycoprotein gene
CI: confidence interval
OR: odds ratio
SNP: single-nucleotide polymorphism
MLG: multilocus genotype
IL: interleukin
NFATP: the National Free Antiretroviral Therapy Programme

## References

[1] Feng Y, Xiao L (2011). Zoonotic potential and molecular epidemiology of *Giardia* species and giardiasis. Clin Microbiol Rev..

[2] Beatriz L, Isabel L, Cristina A, Soledad F, Julio T, Del Aguila C (2002). Intestinal microsporidiosis due to *Enterocytozoon bieneusi* in elderly human immunodeficiency virus-negative patients from Vigo, Spain. Clin Infect Dis..

[3] Xiao L (2010). Molecular epidemiology of cryptosporidiosis: an update. Exp Parasitol..

[4] Li J, Qi M, Chang Y, Wang R, Li T, Dong H (2015). Molecular characterization of *Cryptosporidium* spp, *Giardia duodenalis*, and *Enterocytozoon bieneusi* in captive wildlife at Zhengzhou Zoo, China. J Eukaryot Microbiol..

[5] Wang R, Li J, Chen Y, Zhang L, Xiao L (2018). Widespread occurrence of *Cryptosporidium* infections in patients with HIV/AIDS: epidemiology, clinical feature, diagnosis, and therapy. Acta Trop..

[6] Čondlová Š, Horčičková M, Sak B, Květoňová D, Hlásková L, Konečný R (2018). *Cryptosporidium apodemi* sp. n. and *Cryptosporidium ditrichi* sp. n. (Apicomplexa: Cryptosporidiidae) in *Apodemus* spp. Eur J Protistol..

[7] Cacciò SM, Lalle M, Svärd SG (2018). Host specificity in the *Giardia duodenalis* species complex. Infect Genet Evol..

[8] Lebbad M, Petersson I, Karlsson L, Botero-Kleiven S, Andersson JO, Svenungsson B (2011). Multilocus genotyping of human *Giardia* isolates suggests limited zoonotic transmission and association between assemblage B and flatulence in children. PLoS Negl Trop Dis..

[9] Li W, Feng Y, Santin M (2019). Host specificity of *Enterocytozoon bieneusi* and public health implications. Trends Parasitol..

[10] Li J, Wang H, Wang R, Zhang L (2017). *Giardia duodenalis* infections in humans and other animals in China. Front Microbiol..

[11] Qiu L, Xia W, Li W, Ping J, Ding S, Liu H (2019). The prevalence of microsporidia in China: a systematic review and meta-analysis. Sci Rep..

[12] Wang S, Wang R, Fan X, Liu T, Zhang L, Zhao G (2018). Prevalence and genotypes of *Enterocytozoon bieneusi* in China. Acta Trop..

[13] Hung CC, Tsaihong JC, Lee YT, Deng HY, Hsiao WH, Chang SY (2007). Prevalence of intestinal infection due to *Cryptosporidium* species among Taiwanese patients with human immunodeficiency virus infection. J Formos Med Assoc..

[14] Mahmoudi MR, Ongerth JE, Karanis P (2017). *Cryptosporidium* and cryptosporidiosis: the Asian perspective. Int J Hyg Environ Health..

[15] Wang L, Xiao L, Duan L, Ye J, Guo Y, Guo M (2013). Concurrent infections of *Giardia duodenalis*, *Enterocytozoon bieneusi*, and *Clostridium difficile* in children during a cryptosporidiosis outbreak in a pediatric hospital in China. PLoS Negl Trop Dis..

[16] Zhang X, Wang Z, Su Y, Liang X, Sun X, Peng S (2011). Identification and genotyping of *Enterocytozoon bieneusi* in China. J Clin Microbiol..

[17] Jiang J, Alderisio KA, Xiao L (2005). Distribution of *cryptosporidium* genotypes in storm event water samples from three watersheds in New York. Appl Environ Microbiol..

[18] Xiao L, Morgan UM, Limor J, Escalante A, Arrowood M, Shulaw W (1999). Genetic diversity within *Cryptosporidium parvum* and related *Cryptosporidium* species. Appl Environ Microbiol..

[19] Essid R, Chelbi H, Siala E, Bensghair I, Menotti J, Bouratbine A (2017). Polymorphism study of *Cryptosporidium hominis* gp60 subtypes circulating in Tunisia. Microb Pathog..

[20] Appelbee AJ, Frederick LM, Heitman TL, Olson ME (2003). Prevalence and genotyping of *Giardia duodenalis* from beef calves in Alberta, Canada. et Parasitol..

[21] Sulaiman IM, Fayer R, Bern C, Gilman RH, Trout JM, Schantz PM (2003). Triosephosphate isomerase gene characterization and potential zoonotic transmission of *Giardia duodenalis*. Emerg Infect Dis..

[22] Cacciò SM, Beck R, Lalle M, Marinculic A, Pozio E (2008). Multilocus genotyping of *Giardia duodenalis* reveals striking differences between assemblages A and B. Int J Parasitol..

[23] Lalle M, Pozio E, Capelli G, Bruschi F, Crotti D, Cacciò SM (2005). Genetic heterogeneity at the beta-giardin locus among human and animal isolates of *Giardia duodenalis* and identification of potentially zoonotic subgenotypes. Int J Parasitol..

[24] Geurden T, Levecke B, Cacciò SM, Visser A, De Groote G, Casaert S (2009). Multilocus genotyping of *Cryptosporidium* and *Giardia* in non-outbreak related cases of diarrhoea in human patients in Belgium. Parasitology..

[25] Sulaiman IM, Fayer R, Yang C, Santin M, Matos O, Xiao L (2004). Molecular characterization of *Enterocytozoon bieneusi* in cattle indicates that only some isolates have zoonotic potential. Parasitol Res..

[26] Al-Delaimy AK, Al-Mekhlafi HM, Nasr NA, Sady H, Atroosh WM, Nashiry M (2014). Epidemiology of intestinal polyparasitism among Orang Asli school children in rural Malaysia. PLoS Negl Trop Dis..

[27] Al-Mohammed HI, Amin TT, Aboulmagd E, Hablus HR, Zaza BO (2010). Prevalence of intestinal parasitic infections and its relationship with socio-demographics and hygienic habits among male primary school children in Al-Ahsa, Saudi Arabia. Asian Pac J Trop Med..

[28] Zheng H, He J, Wang L, Zhang R, Ding Z, Hu W (2018). Risk factors and spatial clusters of *Cryptosporidium* infection among school-age children in a rural region of eastern China. Int J Env Res Public Heal..

[29] Conigliaro P, Triggianese P, De Martino E, Chimenti MS, Sunzini F, Viola A (2019). Challenges in the treatment of rheumatoid arthritis. Autoimmun Rev..

[30] Zhang SX, Zhou YM, Xu W, Tian LG, Chen JX, Chen SH (2016). Impact of co-infections with enteric pathogens on children suffering from acute diarrhea in southwest China. Infect Dis Poverty..

[31] Feng Y, Wang L, Duan L, Gomez-Puerta LA, Zhang L, Zhao X (2012). Extended outbreak of cryptosporidiosis in a pediatric hospital, China. Emerg Infect Dis..

[32] Wang T, Fan Y, Koehler AV, Ma G, Li T, Hu M (2017). First survey of *Cryptosporidium*, *Giardia* and *Enterocytozoon* in diarrhoeic children from Wuhan. China. Infect Genet Evol..

[33] Wang L, Zhang H, Zhao X, Zhang L, Zhang G, Guo M (2013). Zoonotic *Cryptosporidium* species and *Enterocytozoon bieneusi* genotypes in HIV-positive patients on antiretroviral therapy. J Clin Microbiol..

[34] Yang D, Yang Y, Wang Y, Yang Y, Dong S, Chen Y (2018). Prevalence and risk factors of *Ascaris lumbricoides*, *Trichuris trichiura* and *Cryptosporidium* infections in elementary school children in southwestern China: a school-based cross-sectional study. Int J Env Res Pub Health..

[35] Wang R, Zhang X, Zhu H, Zhang L, Feng Y, Jian F (2011). Genetic characterizations of *Cryptosporidium* spp. and *Giardia duodenalis* in humans in Henan. China. Exp Parasitol..

[36] de Lucio A, Merino FJ, Martinez-Ruiz R, Bailo B, Aguilera M, Fuentes I (2016). Molecular genotyping and sub-genotyping of *Cryptosporidium* spp isolates from symptomatic individuals attending two major public hospitals in Madrid, Spain. Infect Genet Evol..

[37] Alves M, Xiao L, Antunes F, Matos O (2006). Distribution of *Cryptosporidium* subtypes in humans and domestic and wild ruminants in Portugal. Parasitol Res..

[38] Hu S, Liu Z, Yan F, Zhang Z, Zhang G, Zhang L (2017). Zoonotic and host-adapted genotypes of *Cryptosporidium* spp., *Giardia duodenalis* and *Enterocytozoon bieneusi* in dairy cattle in Hebei and Tianjin, China. Vet Parasitol..

[39] Wang R, Wang H, Sun Y, Zhang L, Jian F, Qi M (2011). Characteristics of *Cryptosporidium* transmission in preweaned dairy cattle in Henan, China. J Clin Microbiol..

[40] Zhang W, Wang R, Yang F, Zhang L, Cao J, Zhang X (2013). Distribution and genetic characterizations of *Cryptosporidium* spp. in pre-weaned dairy calves in northeastern China’s Heilongjiang Province. PloS One..

[41] Wang R, Zhao G, Gong Y, Zhang L (2017). Advances and perspectives on the epidemiology of bovine *Cryptosporidium* in China in the past 30 years. Front Microbiol..

[42] Li F, Wang H, Zhang Z, Li J, Wang C, Zhao J (2016). Prevalence and molecular characterization of *Cryptosporidium* spp and *Giardia duodenalis* in dairy cattle in Beijing, China. Vet Parasitol..

[43] Jian F, Liu A, Wang R, Zhang S, Qi M, Zhao W (2016). Common occurrence of *Cryptosporidium hominis* in horses and donkeys. Infect Genet Evol..

[44] Liu H, Shen Y, Yin J, Yuan Z, Jiang Y, Xu Y (2014). Prevalence and genetic characterization of *Cryptosporidium*, *Enterocytozoon*, *Giardia* and *Cyclospora* in diarrheal outpatients in China. BMC Infect Dis..

[45] Mukherjee AK, Chowdhury P, Rajendran K, Nozaki T, Ganguly S (2014). Association between *Giardia duodenalis* and coinfection with other diarrhea-causing pathogens in India. BioMed Res Int..

[46] de Lucio A, Amor-Aramendia A, Bailo B, Saugar JM, Anegagrie M, Arroyo A (2016). Prevalence and genetic diversity of *Giardia duodenalis* and *Cryptosporidium* spp among school children in a rural area of the Amhara region, north-west Ethiopia. PloS ONE..

[47] Yu F, Amer S, Qi M, Wang R, Wang Y, Zhang S (2019). Multilocus genotyping of *Giardia duodenalis* isolated from patients in Egypt. Acta Trop..

[48] Naguib D, El-Gohary AH, Roellig D, Mohamed AA, Arafat N, Wang Y (2018). Molecular characterization of *Cryptosporidium* spp and *Giardia duodenalis* in children in Egypt. Parasit Vectors..

[49] Yang J, Song M, Wan Q, Li Y, Lu Y, Jiang Y (2014). *Enterocytozoon bieneusi* genotypes in children in northeast China and assessment of risk of zoonotic transmission. J Clin Microbiol..

[50] Zhang W, Ren G, Zhao W, Yang Z, Shen Y, Sun Y (2017). Genotyping of *Enterocytozoon bieneusi* and subtyping of *Blastocystis* in cancer patients: relationship to diarrhea and assessment of zoonotic transmission. Front Microbiol..

[51] Liu H, Jiang Z, Yuan Z, Yin J, Wang Z, Yu B (2017). Infection by and genotype characteristics of *Enterocytozoon bieneusi* in HIV/AIDS patients from Guangxi Zhuang autonomous region, China. BMC Infect Dis..

[52] Yu F, Qi M, Zhao Z, Lv C, Wang Y, Wang R (2019). The potential role of synanthropic rodents and flies in the transmission of *Enterocytozoon bieneusi* on a dairy cattle farm in China. J Eukaryot Microbiol..

[53] Karim MR, Dong H, Li T, Yu F, Li D, Zhang L (2015). Predomination and new genotypes of *Enterocytozoon bieneusi* in captive nonhuman primates in zoos in China: high genetic diversity and zoonotic significance. PloS ONE..

[54] Li J, Luo N, Wang C, Qi M, Cao J, Cui Z (2016). Occurrence, molecular characterization and predominant genotypes of *Enterocytozoon bieneusi* in dairy cattle in Henan and Ningxia, China. Parasit Vectors..

[55] Qi M, Wang R, Wang H, Jian F, Li J, Zhao J (2016). *Enterocytozoon bieneusi* genotypes in grazing horses in China and their zoonotic transmission potential. J Eukaryot Microbiol..

